# Differentially expressed long noncoding RNAs and mRNAs in PC12 cells under lysophosphatidylcholine stimulation

**DOI:** 10.1038/s41598-022-21676-5

**Published:** 2022-11-11

**Authors:** Wen Zhang, Su Dun, Yin Ping, Qingliang Wang, Siqin Tana, Aodong Tana, Si Qin, Xilinqiqige Bao, Alateng Qimuge, Tegexi Baiyin, Dezhi Yang, Siqin Bao, Seyin Baoyin, Wuhan Qimuge

**Affiliations:** 1 School of Life Science, Inner Mongolia University, Hohhot, 010021 China; 2 Research and Development Center, HUA Cloud Intelligent Healthcare Co., Ltd, Shenzhen, 518000 China; 3 Affiliated Hospital of Inner Mongolia University for Nationalities, Tongliao, 028000 China; 4 Inner Mongolia Medical University, Hohhot, 010010 China; 5 Inner Mongolia International Mongolian Hospital, Hohhot, 010065 China; 6 Inner Mongolia Traditional Chinese&Mongolian Medical Research Institute, Hohhot, 010017 China

**Keywords:** Biochemistry, Cell biology, Genetics, Neurology

## Abstract

Lysophosphatidylcholine (LPC) was previously found to show neuroprotective effect on nerve growth factor (NGF) and brain derived neurotrophic factor (BDNF) induced signalings. Also, numerous studies reported the emerging roles of long noncoding RNAs (LncRNAs) involved in neurodegenerative disease. However, the biological mechanism of LPC and expression profile of lncRNAs has not been reported. Here, lncRNAs in PC12 cells under LPC and NGF treatment were analyzed using high throughput sequencing technology for the first time. We identified 564 annotated and 1077 novel lncRNAs in PC12 cells. Among them, 121 lncRNAs were differentially expressed in the PC12 cells under LPC stimulation. KEGG analysis showed that differentially expressed mRNAs co-expressed with lncRNAs mainly enriched in ribosome, oxidative phosphorylation, Parkinson’s disease, Huntington’s disease and Alzheimer’s disease etc. LncRNA-mRNA network analysis showed that lncRNA ENSRNOT00000082515 had interactions with 626 different mRNAs suggesting that lncRNA ENSRNOT00000082515 probably play vital role. Finally, sequencing data were validated by qRT-PCR for ENSRNOT00000084874, ENSRNOT00000082515, LNC_001033 forward *Fgf18*, *Vcam1*, and *Pck2*.

## Introduction

Alzheimer's disease (AD) is the most common form of dementia. This neurodegenerative disease mainly occurs in older people, but some cases tend to happen in the young. The early symptoms of patients with AD are the impairment in short term memory. Progressively the patients get difficulty in language and daily activities. Eventually, people lose the long-term memory and cannot recognize their families^1–3^. The development of AD was mainly characterized by β-amyloid (Aβ) plague, neurofibrillary tangles (NFTs) and neuronal loss. Aβ protein is the main protein of brain plaques and is mainly produced from amyloid precursor protein (APP). β-site amyloid precursor protein cleaving enzyme 1(BACE1) is one of the essential enzyme to produce Aβ peptides from APP. Hence, BACE1 is an attractive target to inhibit the Aβ secretion^4–7^. In normal brain, tau protein interacts with tubulin/microtubules to support the stable and flexible state of axonal microtubules. It also interacts with protein kinase Fyn suggesting some important role in Fyn signaling. Besides, the interaction of tau with actin was reported. But in patient with AD, tau protein is abnormally hyperphosphorylated and has important role in NFTs. Presently, Mitogen-activated protein (MAP) kinase, glycogen synthase kinase-3 (GSK-3), cyclin-dependent kinase-5 (Cdk5), Cyclic adenosine monophosphate (AMP)-dependent protein kinase A (PKA), protein kinase B (PKB) and microtubule-affinity regulating kinase (MARK) have been found to phosphorylate tau in vitro^4,5,8^.

Long noncoding RNA (lncRNA)s are transcription longer than 200 that are not translated into protein. Recently, it was found that some of lncRNAs encodes proteins. lncRNAs present in the nucleus of various type of cells and are involved in many cellular processes. lncRNAs function in the inactivation of X-chromosome, imprinting, development and antiviral response of genes and the transcriptional dynamics of nuclear speckles. lncRNAs also act in the post-transcriptional process of proteins. Furthermore, lncRNAs were found to be associated with various diseases, such as cancer, cardiovascular diseases, genetic diseases and neurodegenerative diseases^9–13^. Increasing evidences have suggested that long non-coding RNAs have been revealed to play important roles in AD pathophysiology through mediating the expression of genes and proteins, such as mediating amyloid beta production, expression level of c-fos and BDNF gene, and neuronal plasticity. One of the widely defined AD-related lncRNA is *BACE1-AS*, which can regulate the expression of BACE1 at both genetic and translational levels. There are lncRNAs *BC200, 17A, 51A, NDM29* were also found to be dysregulated in AD and affect the pathway of Aβ production^1^. Increasing evidences have proved the emerging roles of lncRNAs in AD and other neurodegenerative diseases. Therefore, lncRNAs involved in neurodegenerative diseases still need to be extensively identified^14–17^.

Lysophosphatidylcholine (LPC) is one of the most abundant Lysophophalipid, known as a bioactive lipid released from the PC of cell membrane or PC of lipoprotein by the hydrolytic activity of sPLA_2_ in the blood^18–20^. LPC can also be generated by endothelial lipase or by lecithin-cholesterol acyltransferase secreted from the liver^18–22^. LPC has been found to show neuroprotective activity by protecting cerebellar granule cells from apoptosis^23^.

LPC modulate various cellular process, such as attracting phagocytes to apoptotic cells by inducing the expression of vascular cell adhesion molecule-1 and intercellular adhesion molecule-1^24,25^, and inducing chemotaxis in other types of cells^26,27^. In addition, LPC was found to increase intracellular Ca^2+^ in macrophages and neurotrophils^28,29^, and activate protein kinase C, activator protein-1, and c-Jun N-terminal kinase in several types of cells^30–33^. By evoking various cellular responses as mentioned above, LPC plays critical roles in sepsis, rheumatoid arthritis and multiple sclerosis, but the mechanisms seem to be complicated. Our previous studies have demonstrated that LPC shows neurotrophin-like activity, induction of neurite outgrowth in PC12 cells and protection of cerebellar granule neurons from apoptosis. In further study, NGF significantly triggered MAPK phosphorylation in PC12 cells, while the treatment with LPC alone (C16:0; 0.1 and 1 μM) failed to induce MAPK phosphorylation. Interestingly, LPC has significantly enhanced NGF-induced MAPK and Akt signaling pathways through TrkA phosphorylation in PC12 cells. Also, LPC potentiated BDNF-induced TrkB phosphorylation and it’s down stream signals in cerebellar granule neurons^34,35^. A recent study also reported that phosphatidylcholine, LPC and phosphatidylserine etc. exhibited neuroprotective effect against corticosterone-induced cytotoxicity in primary cultured rat cortical neurons^36^.

There is a potential role for LPC in treating AD, not only because of its neuroprotective effect itself, also because of that it was found to enhance the transportation of DHA into brain through blood brain barrier (BBB) and improve memory. LPC was recently found to be a preferred carrier for PUFA across BBB into brain. These findings indicate that LPC play an important role in the pathology of AD^37–39^.

Although the neurotrophin like effect of LPC in PC12 cells and cerebellar neuron cells was indicated in our previous study, few studies to date, however, have evaluated lncRNAs expression profile in PC12 cells and whether or not lncRNAs are involved in the neutrohin-like activity of LPC in PC12 cells. Hence, in this study, the expression profiles of both mRNAs and lncRNAs in PC12 cells under different stimulations were detected by adopting Illumina Hiseq 4000 sequencing platform. Differentially expressed mRNAs and lncRNAs were screened and their biological functions were predicted.

## Results

### Total RNA quality control

Four sets of PC12 cells were untreated or treated with NGF and LPC either alone or in combination at specified concentration for 30 min. Then, the total RNA was extracted from cells and was proceeded for quality control test. The results showed that the purity (A260/A280) of total RNA from all samples was around 2.0, and the bands of RNA on the gel after electrophoresis were clear, indicating that there were no proteins, genomic contamination, and other contamination. Thus, the total RNA from all samples was fit for further analysis.

### Genome-wide identification of lncRNAs and mRNAs in PC12 cells

A total of 1.156765658 billion raw reads from 12 samples were generated by Illumina HiSeq 4000 Platform. The raw reads were filtered by removing mistakes and contaminations. Consequently, a total of 1.092943262 billion clean reads were obtained. Subsequently, the clean reads were aligned to the rat reference genome (ftp://ftp.ensembl.org/pub/release-86/fasta/rattus_norvegicus/dna), and the average mapping rate of samples from rat cells stimulated by control, LPC, NGF, and LPC plus NGF was 88.20, 88.90, 88.86 and 88.41%, respectively. Through cufflinks assembles results from the TopHat2 alignments, a total of 100,133 transcripts were finally obtained and used for subsequent analysis.

**Figure 1:**
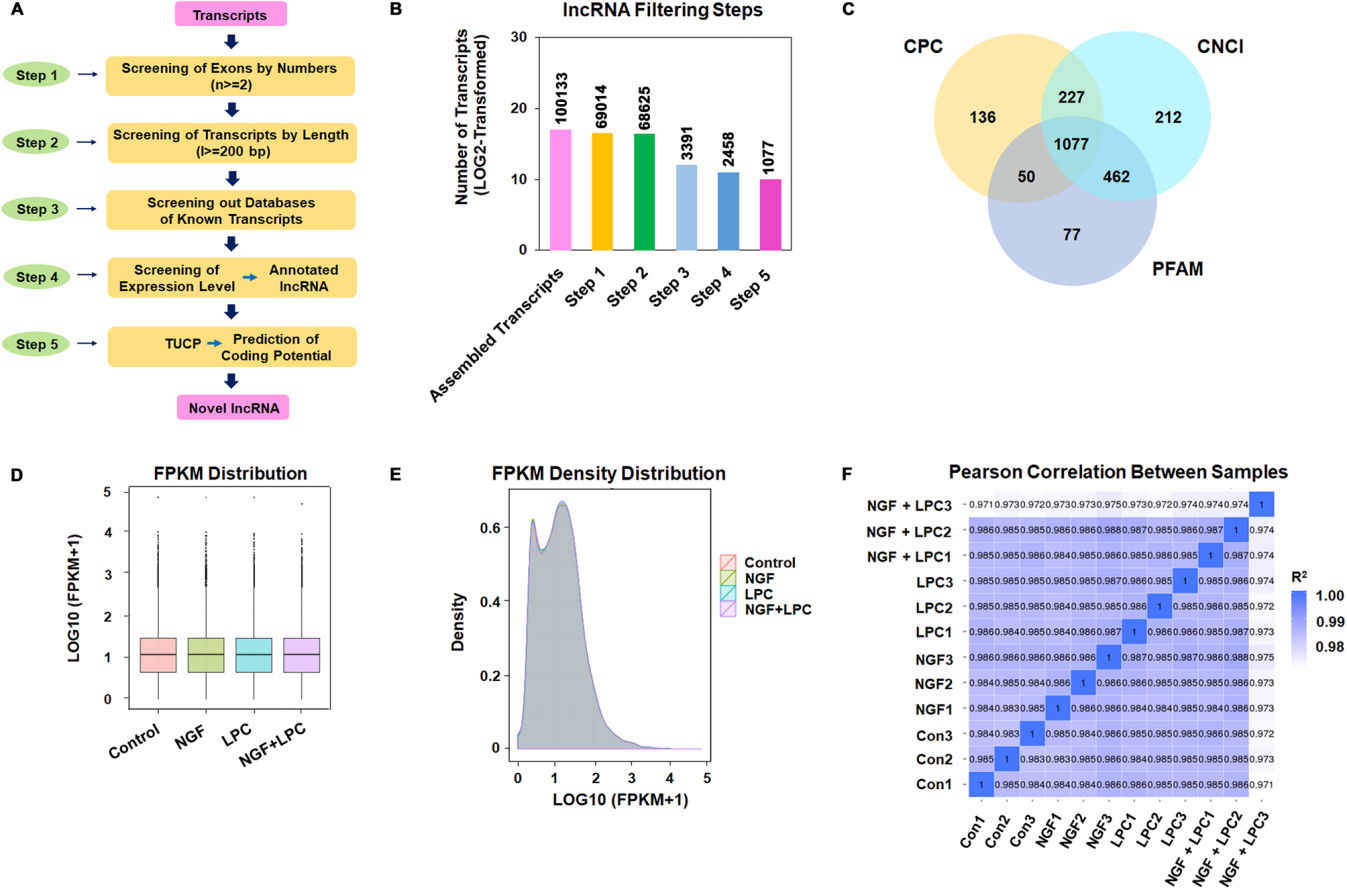
Identification of lncRNAs in PC12 cell lines. (**A**) Flow chart of lncRNAs analysis; (**B**) Histogram representation of lncRNAs filtration; (**C**) Venne diagram of novel lncRNAs predicted based on CPC, CNCI and PFAM; (**D**) FPKM distribution analysis among four experimental groups; (**E**) FPKM density analysis among four experimental groups; (**F**) Pearson coefficient analysis four experimental groups.

In order to minimize the false positive rates in identifying lncRNAs from 100,133 of assembled transcripts collected from all four group samples, a stringent filtering outline were developed to remove the transcripts without all features of lncRNAs (Fig. 1A). After screening out the transcripts which were less than two exons, 200 bp in length, three reads coverage and 0.5 FPKM value, and 2458 of novel lncRNAs were found. Then, the remained transcripts were blasted with known classes of RNAs and protein-coding genes, and finally 1077 novel lncRNAs were identified by CPC, CNCI and PFAM prediction (Fig. 1B, C).

To test the effectiveness of data and irrationality of experiment design, the expected number of fragments per kilobase of transcripts sequence per million base pairs sequenced (FRKM) and Pearson correlation of all detected novel lncRNAs and annotated lncRNAs from all groups of cells were analyzed by comparing to those in mRNAs. FRKM distribution (Fig. 1D) and density distribution (Fig. 1E) results under different experimental conditions showed that the expression level of different samples were similar. The R^2^ values of Pearson correlations between each sample were higher than 0.92 (Fig. 1F), indicating that the RNA-seq data from each sample were reliable.

### Genome characteristics of lncRNAs in PC12 cells

To examine the molecular difference of lncRNAs and mRNAs, and verify whether lncRNAs match the general features, we analyzed the length, exon, and ORF length (EMBOSS:getorf) of annotated lncRNAs and novel lncRNAs from all groups of cells and made a comparison with mRNAs.

**Figure 2:**
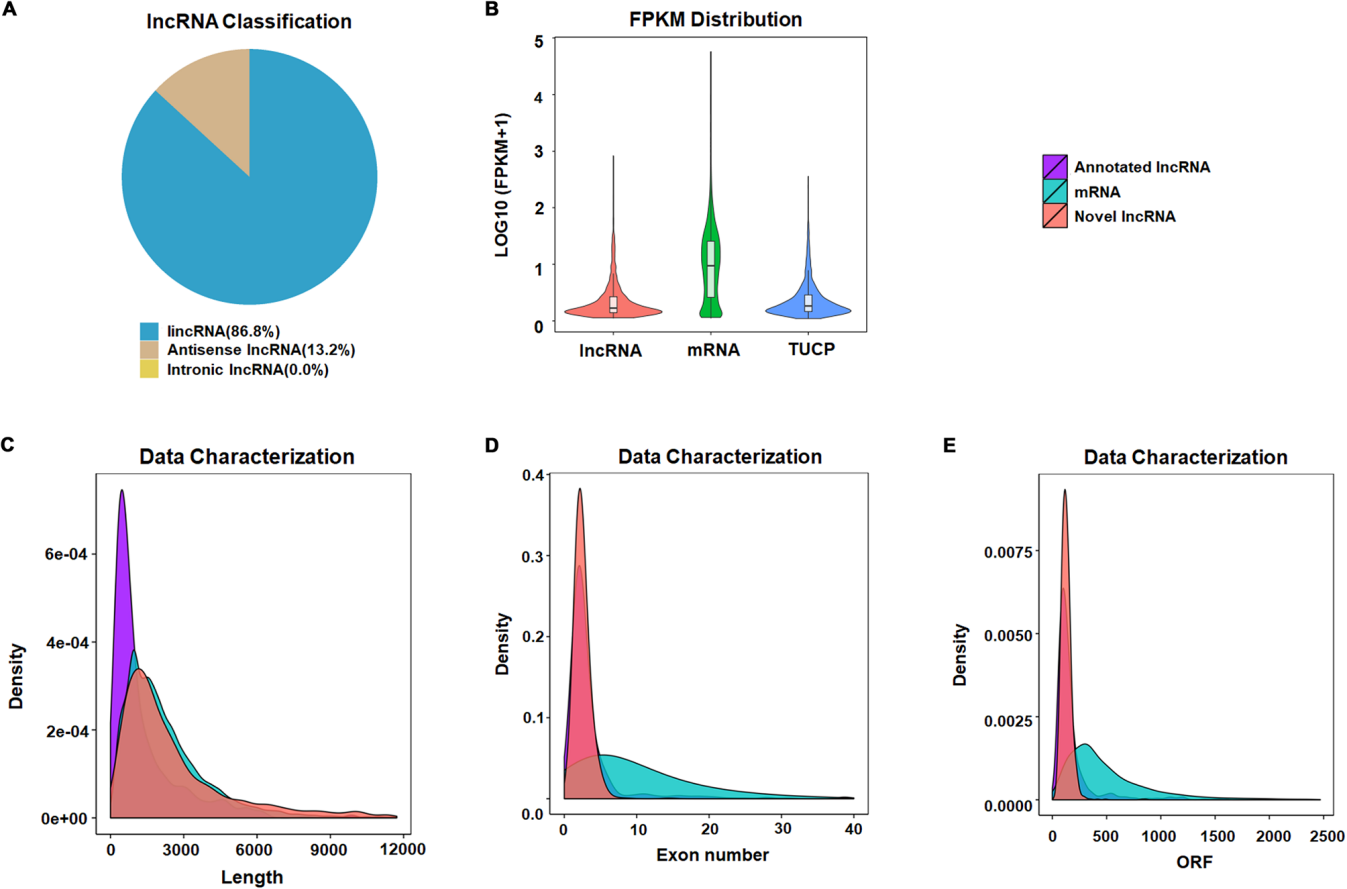
Genomic characterization of lncRNAs in PC12 cell lines. (**A**) A pie chart illustrating the classification of lncRNAs derived from PC12; (**B**) Expression level distribution of lncRNAs and mRNAs derived from PC12 cells; (**C**) Representation of the transcript length distribution derived from PC12 cells; (**D**) Exon numbers of transcripts derived from PC12 cells; (**E**) Open reading frame (ORF) of transcripts derived from PC12 cells.

The novel lncRNAs contained 86.8% of lincRNAs and 13.2% of anti-sense lncRNAs (Fig. 2A). As shown in (Fig. 2B), the expression level of lncRNAs is generally lower than that of mRNAs. Certain part of the transcripts was found to have uncertain coding potential (TUCP). They shared similar expression level with lncRNAs. The most annotated lncRNAs were shorter than 1500 bp, while novel lncRNAs were distributed in the similar region with mRNAs less than 5000 bp, and a small part of novel lncRNAs had longer base-pairing length of 5000 ~ 12,000 bp (Fig. 2C). Generally, annotated and novel lncRNAs had less exons compared to mRNAs (Fig. 2D). In addition, both annotated lncRNAs and novel lncRNAs had shorter open reading frame (ORF) than mRNAs (Fig. 2E).

### Gene enrichment and KEGG pathway analysis of lncRNAs in serum-starved PC12 cells

**Figure 3:**
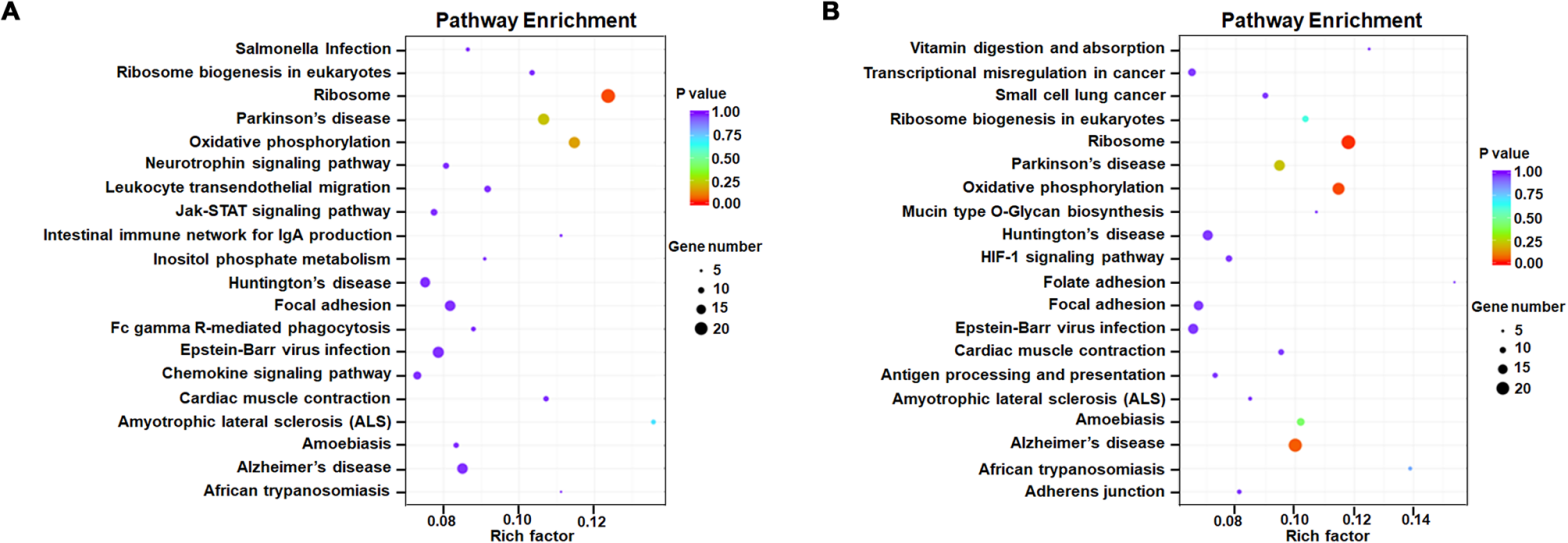
Pathway enrichment of lncRNAs co-expressed genes derived from PC12. (**A**) KEGG pathway analysis of the co-expressed genes of the annotated lncRNAs; (**B**) KEGG pathway analysis of the co-expressed genes of the novel lncRNAs. The horizontal axis represents enrichment factor and the vertical axis represents pathway. Different colors represent different adjusted *p* values. From purple to red, the adjusted *p* value changes from large to small and the degree of enrichment becomes more and more significant. The size of the dot represents the number of genes enriched into this pathway.

To further understand the potential role of lncRNAs in PC12 cells, biological roles were predicted by KEGG enrichment analysis for annotated and novel lncRNAs through co-expression and co-located genes, respectively. Here, we focused on the KEGG pathway analysis conducted by co-expressed genes. The genes co-expressed with annotated lncRNAs are mainly enriched in ribosome, Parkinson’s disease (PD), oxidative phosphorylation, Huntington’s disease (HD), focal adhesion, Epstein-Barr virus infection, and AD as shown in Fig. 3A. Interestingly, the genes co-expressed with novel lncRNAs found in this study were also enriched in ribosome, oxidative phosphosylation, AD, PD and HD etc. (Fig. 3B).

### mRNAs and LncRNAs expression profiles in PC12 cells under treatment of LPC and NGF, either alone or together

In order to analyze lncRNAs differentially expressed under the treatment of LPC or NGF, lncRNAs were examined in PC12 cells cultured with addition of either LPC or NGF alone or both together.

In PC12 cells stimulated by LPC, there were 483 mRNAs differentially expressed, that included 226 up-regulated and 257 down-regulated (Supplementary Table 1), while there were 121 lncRNAs differentially expressed, that included 45 up-regulated and 76 down-regulated compared to those in control group (Supplementary Table 2).

In PC12 cells stimulated by NGF, 555 mRNAs differentially expressed, including 283 up-regulated and 272 down-regulated (Supplementary Table 3), while 122 lncRNAs differentially expressed, with 26 up-regulated and 96 down-regulated compared to those in control group (Supplementary Table 4).

We also compared the expression level of mRNAs and lncRNAs in the group under LPC plus NGF treatment with control group, and found the expression level of 1214 mRNAs were significantly regulated, in which, 608 up regulated and 606 down regulated (Supplementary Table 5); the expression level of 227 lncRNAs were significantly regulated, in which, 38 up regulated and 189 down regulated (Supplementary Table 6).

When cells were treated with NGF plus LPC, the expression of 941 mRNAs and 111 lncRNAs were significantly regulated compared to those in cells under only NGF treatment (Supplementary Tables 7 and 8). When the expression profile of mRNA and lncRNA under NGF or LPC compared, 529 mRNAs and 91 lncRNAs were found to be differentially expressed (Supplementary Tables 9 and 10).

**Figure 4:**
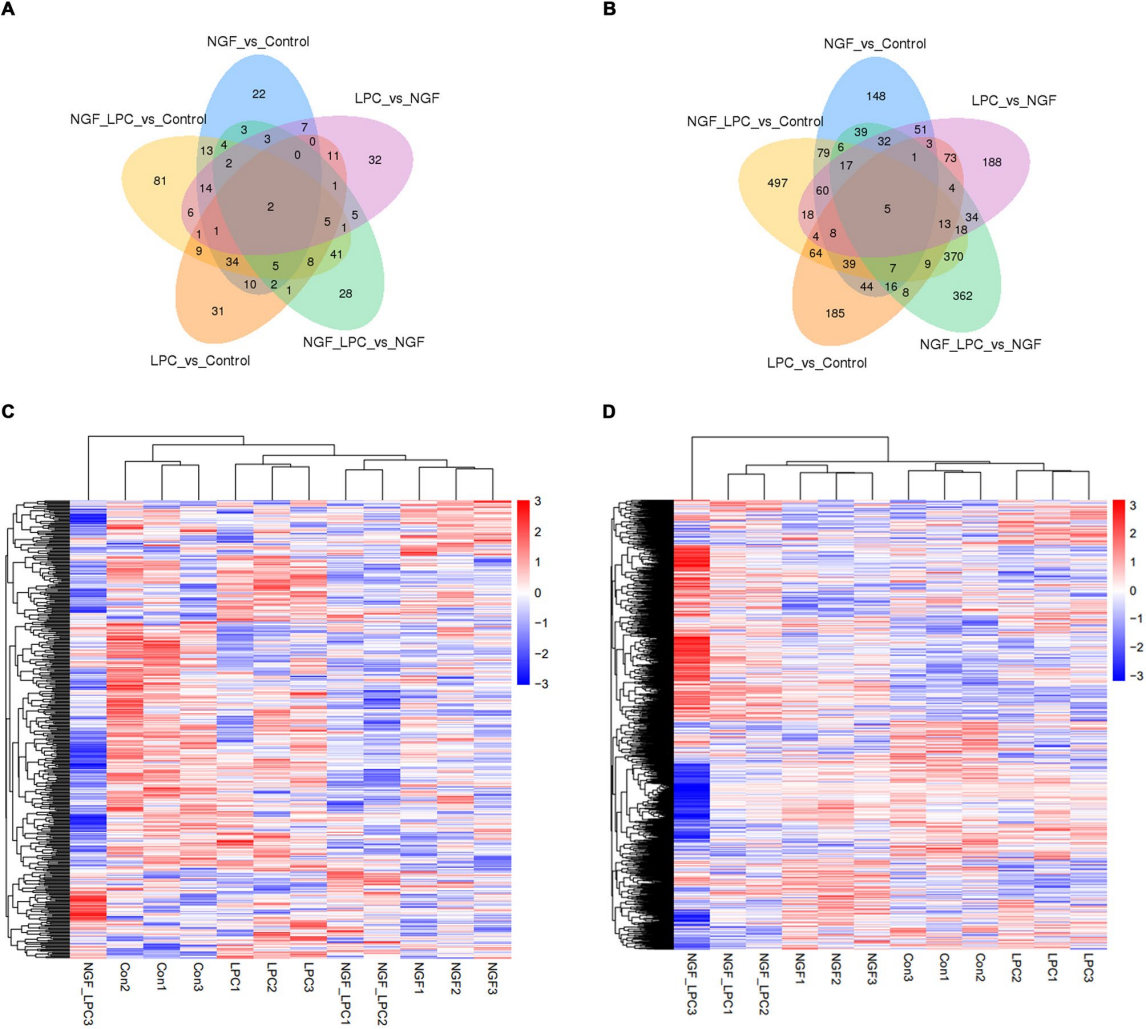
mRNAs and lncRNAs profiles from cells under different treatments and showed in Venn diagram and Heatmap analysis. (**A**, **B**): Venn diagram for mRNA and lncRNA pieceful. (**C**, **D**): Heatmap analysis for all differentially expressed mRNAs and lncRNAs.

Various mRNAs and lncRNAs were expressed differentially in cells under different treatments (Fig. 4A and B). The differentially expressed mRNAs and lncRNAs were then applied to a systematic heatmap cluster (Fig. 4C and D). Details can be found in Supplementary Tables 1–10. Besides, further analysis found that differentially expressed lncRNAs transcripts almost distributed through all chromosomes.It suggests that LPC and/or NGF mediate various lncRNAs in PC12 cells, which might involve their biological functions.

### Prediction of target genes and lncRNA-mRNA network analysis

Generally, lncRNAs play biological roles through co-expressed or co-localized protein-coding genes. Here, co-localized genes with lncRNAs were predicted through detecting the genes on 100 kb downstream or upstream of lncRNAs (data not shown). Also, co-expressed genes with lncRNAs were analyzed by screening the genes with Pearson correlation > 0.95.

**Figure 5:**
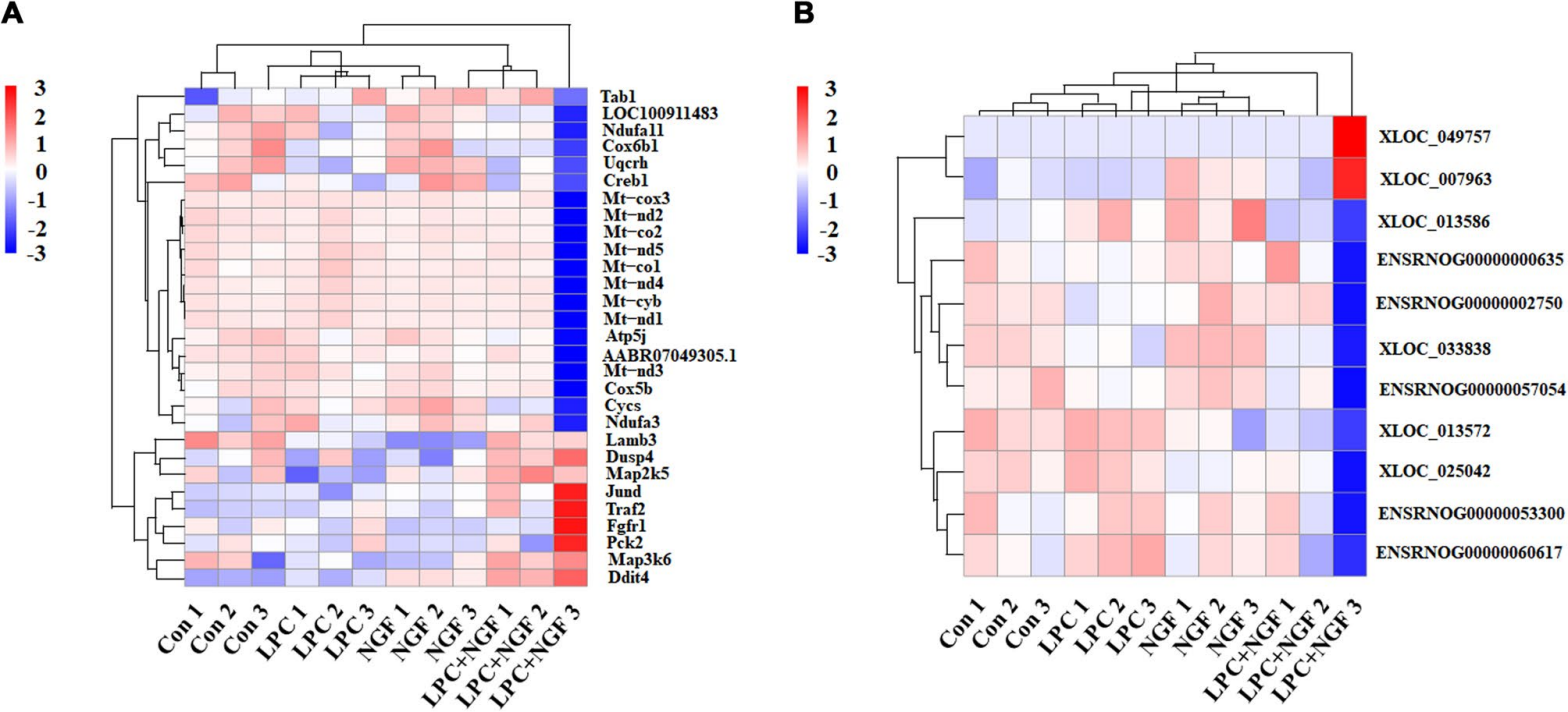
Co-expression network of lncRNAs and mRNAs in PC12. The squared nodes represent lncRNAs, whereas the circled nodes represent mRNAs; the node size is denoted based on degree (higher values are mapped to the bright color) and the edge is denoted based on the Pearson values.

Furthermore, we analyzed the target interactions based on co-expressed lncRNAs and mRNAs data. As shown in Fig. 5 the network profile showed 766 nodes and 3392 connections between annotated and novel lncRNAs and their targets mRNAs. It was found that one lncRNAs might have one or more target genes; suggesting one lncRNA is possible to regulate and function on multiple genes. Reversely, numerous genes might be mediated by single lncRNA. Here, annotated lncRNA ENSRNOT00000082515 was found to have the maximum interactions with 626 mRNAs. Also, novel lncRNA LNC_001033 showed connections with 313 mRNAs. Annotated lncRNA ENSRNOT00000084874 showed interactions with 122 genes. These results indicated that lncRNAs ENSRNOT00000082515 (ENSRNOG00000061060), LNC_001033 (XLOC_049757) and ENSRNOT00000084874 (ENSRNOG00000056832) have the most active potential among them and probably regulate the expression of various genes.

It was previously found that LPC promotes NGF-induced MAPK and Akt signaling cascades through enhancing NGF-induced MAPK and Akt phosphorylation, which are known to be essential for the differentiation and survival of neurons, as the consequence of enhanced activation of the receptor TrkA in PC12 cells. But, LPC itself didn’t induce MAPK and Akt phosphorylation. To identify the genes involved in the neurotrophin-like activity of LPC, mRNAs up-regulated in MAPK and PI3K-Akt pathways under NGF plus LPC treatment were compared to that under NGF stimulation. There were 9 genes (17 transcripts) up-regulated under the condition of LPC and NGF treatment: ENSRNOG00000002244; ENSRNOG00000011921; ENSRNOG00000019568; ENSRNOG00000017285; ENSRNOG00000006238; ENSRNOG00000007926; ENSRNOG00000008936; ENSRNOG00000016050; ENSRNOG00000006612, with KO names as follows: Pdgfra, APDGFR, PDGFACE; Dusp4, Mkp-2, Mkp2; Jund; Tab1, Map3k7ip1; Traf2; Map2k5, Mek5; Map3k6; Fgfr1, respectively. LPC seems mediate different set of genes (483 mRNAs) when stimulate cells alone compared with untreated cells.

### Validation of the sequencing data by qRT-PCR

**Figure 6:**
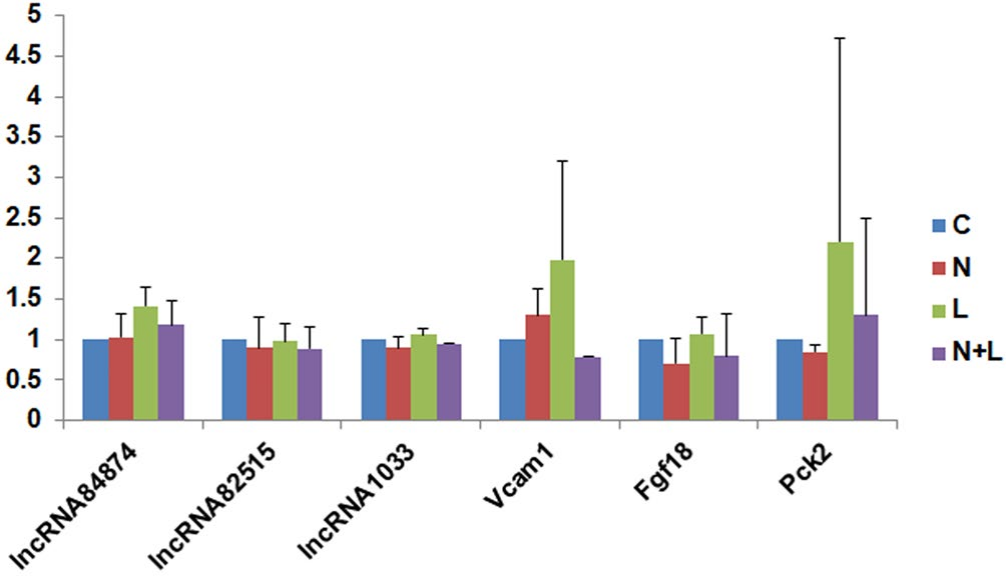
qRT-PCR validation of the sequencing results. The data showed that the relative expression level of genes *fgf18*, *Pck2*, *Vcam1*, *Wnt5a* and *Fos* in sequencing (black) and qRT-PCR experiments (gray). The relative expression level of genes here represents the fold change of genes by normalizing the 2^−ΔΔCT^ value of each group by that of control group. The relative expression of *fgf18*, *Pck2* and *Vcam1* in sequencing and qRT-PCR were consistent: the expression of *fgf18* and *Pck2* were significantly up-regulated, while the expression of *Vcam1* was down regulated (*p* < 0.05).

In order to validate our sequencing results, ENSRNOT00000084874, ENSRNOT00000082515, LNC_001033, *Fgf18*, *Vcam1*, and *Pck2* differentially expressed genes were selected. The qPCR results of *Fgf18*, *PCK2* and *Vcam1* were consistent with the sequencing results. In addition, compared with the control group and NGF group, the expression of *Fgf18* was significantly increased in LPC and NGF plus LPC group respectively, while the expression of *Vcam1* was significantly decreased in the LPC group (Fig. 6).

Fibroblast growth factor 18 (*Fgf18*) has been found to be involved in midbrain development and protecting neurons from ischemic injury. Further research indicated that *Fgf18* showed a potential role for PD therapy and Osteoarthritis^40,41^. In current study, expression of gene *Fgf18*was significantly increased by LPC compared to the control suggests that a potential role of LPC in the protection of neurons through regulating *Fgf18*.

Vascular cell adhesion molecule-1 (*Vcam1*) was a cell adhesion molecule that involves regulating inflammation-associated vascular adhesion and the trans-endothelial migration of leukocytes, such as macrophages and T cells. It was found by recent researches that *Vcam1* plays very important roles in the progression of various immunological disorders, including rheumatoid arthritis, asthma, transplant rejection, and cancer^42^. Here, the expression of *Vcam1* was down-regulated by LPC.

## Discussion

Rat pheochromocytoma PC12 cell line is a suitable model for studying the cellular responses of growth factors at the molecular level^42,43^. Increasing numbers of studies have indicated that lncRNAs are implicated in various biological processes of neurological diseases through mediating mRNAs^44–47^. However, the expression profile of lncRNAs in PC12 cells has not been reported so far. In this study, besides analyzing mRNAs expression, the lncRNAs expression profiles in PC12 cells under different stimulus were evaluated for the first time. As a result, 564 annotated lncRNAs and 1077 novel lncRNAs were elucidated and their characteristics were further determined. This sequencing results would provide benefical information for further studying lncRNAs’ biological roles in PC12 cells.

The expression of lncRNAs following various stimulations showed different model. 358, 382, 703 of lncRNAs and 556, 486, 1214 of mRNAs under treatment of NGF, LPC, NGF plus LPC respectively, were significantly differentially expressed in PC12 cells (*p* < 0.05).

In our previous work, LPC showed neurotrophin-like activity, such as promoting cell proliferation of PC12 cells and inhibited the apoptosis of cerebellar granule neurons, through enhancing the phosphorylation of MAPK and Akt pathways. Here, *Fgf18* enriched in MAPK and PI3K-Akt signaling pathways through KEGG analysis was upregulated by LPC. Also, the expression of *Pck2* enriched in PI3K-Akt pathway in KEGG analysis was tend to be upreglated by LPC. Reversely, the expression of *Vcam* was downregulated under NGF plus LPC stimulation compared to that under only NGF treatment. The network analysis between lncRNAs and mRNAs indicated that there were three lncRNAs [ENSRNOT00000082515 (ENSRNOG00000061060), LNC_001033 (XLOC_049757) and ENSRNOT00000084874 (ENSRNOG00000056832)]showed most active interaction with differentially expressed mRNAs enriched in AD, PD, and HD (Parkinson’s disease (PD), Huntington’s disease (HD) pathways. These mRNAs were mainly mitocondria related factors. However, the expression of these lncRNAs themselves under different treatment were not significantly altered. Therefore, LncRNAs involve in the expression of mRNAs may not through their co-expression. There might be another function for lncRNAs to affect the expression of mitocondria genes which might play vital roles in AD, PD and HD. This mechanism can be considered as one of the topic in the future research.

It is interesting that one lncRNA can interact with various mRNAs. This result indicates that lncRNA possibly mediate the expression of different mRNAs at the same time.

In present study, we found that LPC regulated many mRNAs’ and LncRNAs’ expression in PC12 cells. In particular, the number of differentially expressed lncRNA was obviously increased when cells were treated by NGF and LPC together. This result indicates that there might be some crosslink between LPC and NGF on the expression of lncRNAs and mRNAs.

Also, 19, 15, and 15 of mRNAs enriched in PD, AD and HD pathways were significantly downregulated by NGF plus LPC stimula compared to that in NGF. Various differentially expressed and most of them were mitocondria related, indicating the potential role of LPC on mintocondria, such as mitochondrially encoded NADH dehydrogenase 1/2/4/5, mitochondrially encoded cytochrome C oxidase III, mitochondrially encoded cytochrome c oxidase II. lncRNA and mRNAs interaction analysis showed ENSRNOG00000031033, ENSRNOG00000030963, ENSRNOG00000031766, ENSRNOG00000030700, ENSRNOG00000029971, ENSRNOG00000030371, ENSRNOG00000030644, ENSRNOG00000034234, ENSRNOG00000029707, the genes including *Fgf18*, *Pck2*, were found to be up-regulated by LPC compared to control. It indicates that LPC might also enhance the activation of MAPK and Akt pathways through mediating the expression of *Fgf18*, *Pck2*.

lncRNAs have been implicated in various neurodegenerative diseases. LncRNAs were found in the late-onset of AD, including *AD-lin1*and *AD-lin2*. The expression level of lncRNA *AD-lin1* increased in Aβ-exposed human neuroal cells and this was supposed to be involved in the amyloid-induced neurotoxicity^17^. In addition, expression of lncRNAs *UCHL1-AS1*, *HTT-AS*, *BDNF-AS* and *HAR1* were found to be upregulated in Parkinson’s disease and Huntington’s disease^1^. Studies carried out in rat model with the hypoxic-ischemic brain damage identifed that lncRNA BC088414 might participate in the neurogenesis since konckdown of it inhibited the cell proliferation and promoted apoptosis of PC12 cells^44^.

Analyzing the emerging roles of lncRNAs in neurodegenerative disease, especially AD could provide new possibilities for understanding the mechanism and potentials for the diagnosis of AD. Since a singular lncRNA is capable to interact with various kinds of mRNAs, this makes lncRNAs functionally important. Understanding the lncRNAs expression profile in PC12 cells would provide vital informations for further research that concerning PC12 cells.

## Materials and methods

### Cell culture

Rat pheochromocytoma PC12 cells (undifferentiated, Zhong Qiao Xin Zhou Biotechnology, Shanghai, China) were maintained in 10 ml of Ham's F-12 K (Kaighn's) medium (F12K) supplemented with 10%FBS, 1%PS, 0.05 mg/ml ascorbic acid; 0.01 mg/ml insulin; 0.01 mg/ml transferrin; 10 ng/ml sodium selenite; 0.03 mg/ml Endothelial Cell Growth Supplement (ECGS) in 10-cm tissue culture dish, at 37 °C in a humidified and CO_2_-controlled (5%) incubator. Cells were kept with regular transfer of once in two or three days. 1 ml of 0.25% trypsin containing 1 mM EDTA was used to detach cells and PBS buffer (pH7.0) was used to wash cells during transfer. PC12 cells were inoculated on collagen type I (rat tail)-coated 6-well culture plates at a density of 4 × 10^5^ cells/well in 2 ml/well F12K, and incubated for 24 h or longer until > 80% confluent. Before cells were subjected to various treatments as specified in the text, culture medium was removed by aspiration. Then, cells were washed twice with serum free F12K medium carefully (do not detach cells), added serum free F12K medium 2 ml/well, and incubated for 1.5 h (serum-starvation). Then, cells were subjected to various treatments as specified in the text. Finally, the collected cells were used for the RNA extraction. 2 wells of cells were prepared and combined after collection for each sample for RNA isolation in each independent experiment. The experiment was repeated three times independently.

### RNA isolation and quality test

Total RNA from cell pallets were isolated by TRIzol Reagent (Invitrogen) in accordance with the manufacturer’s instructions.

Total RNA was qualified and quantified as follows: (1) the RNA sample was firstly qualified using 1% agarose gel electrophoresis for possible contamination and degradation; (2) RNA purity and concentration were then examined using NanoPhotometer® spectrophotometer; (3) RNA integrity and quantity were finally measured using RNA Nano 6000 Assay Kit of the Bioanalyzer 2100 system.

RNA library for lncRNA-seq was prepared as rRNA depletion and stranded method using NEBNext® Ultra™ RNA Library Prep Kit for Illumina®. Briefly, the ribosomal RNA was depleted from total RNA using the rRNA Removal Kit following manufacturer’s instruction. RNA was then fragmented into 250 ~ 300 bp fragments, and first strand cDNA was reverse transcribed using fragmented RNA and dNTPs (dATP, dTTP, dCTP and dGTP). RNA was degraded using RNase H, and second strand cDNA was synthesised using DNA polymerase I and dNTPs (dATP, dUTP, dCTP and dGTP). Remaining overhangs of double-strand cDNA were converted into blunt ends via exonuclease/ polymerase activities. After adenylation of 3’ ends of DNA fragments, sequencing adaptors were ligated to the cDNA. In order to select cDNA fragments of preferentially 250 ~ 300 bp in length, the library fragments were purified with AMPure XP system. Uridine digestion was performed using Uracil-N-Glycosylase, which was followed by the cDNA amplification using PCR.

After library construction, the concentration of library was measured by the Qubit® fluorometer and adjusted to 1 ng/μl. Agilent 2100 Bioanalyzer was deployed to examine the insert size of the acquired library. Once the insert size of lncRNAs were 250–300 bp, and concentration of the library was between 1.5 and 30 nm, it was considered as identical, the samples can then be subjected for sequencing.

### Sequencing

After library preparation and pooling of different samples, the samples were subjected for Illumina sequencing by Illumina HiseqX. Commonly, the lncRNA-seq used PE150 (paired-end 150nt) sequencing for 12G raw data.

### Quality control of sequenced data

Raw sequenced reads of fastq format were firstly processed through in-house perl scripts. In this step, clean reads were obtained by removing reads containing adapter, reads on containing ploy-N and low quality reads from raw data. At the same time, Q20, Q30 and GC content (%) of the clean data were calculated. The clean data with high quality obtained here were used for all the further analyses.

### Mapping to the reference genome and transcriptome assembly

Reference genome and gene model annotation files were downloaded from genome website (ftp://ftp.ensembl.org/pub/release-86/fasta/rattus_norvegicus/dna) directly. Index of the reference genome was built using Bowtie v2.0.6 and paired-end clean reads were aligned to the reference genome using TopHat v2.0.9^48^.

The mapped reads of each sample were assembled by Cufflinks (v2.1.1)^49^ in a reference-based approach. Cufflinks used a probabilistic model to simultaneously assemble and quantify the expression level of a minimal set of isoforms that provided a maximum likelihood explanation of the expression data in a given locus^50^. Cufflinks was run with ‘min-frags-per-transfrag = 0’ and ‘–library-type’, other parameters were set as default.

All the transcripts were merged using Cuffmerge software. LncRNAs were then identified from the assembled transcripts following four steps: (1) Removal of low expressed transcripts with FPKM < 0.5; (2) removal of short transcripts < 200 bp and < 2 exons; (3) removal of the transcripts with protein coding capability using CNCI, Pfam and CPC2 database; (4) removal of the transcripts mapped within 1 kb flanking regions of an annotated gene using Cuff compare. Novel lncRNAs were named following rules of HGNC (The HUGO Gene Nomenclature Committee). The characteristics of novel lncRNAs were compared with known lncRNAs and mRNAs^51^.

### Characteristic analysis of lncRNAs

Screened lncRNAs were classified by lincRNA, anti-sense_lncRNA, intronic_lncRNAs and the percentage was calculated.

The length, exon, and ORF length (EMBOSS:getorf) of annotated lncRNAs , novel lncRNAs were analyzed by comparing to those in mRNAs.

Quantification of the transcripts and genes was performed using StringTie software and Reads Per Kilobase of transcript per Million mapped reads (RPKM) was obtained. Cuffdiff or edgeR was used for differential expression analysis. The resulting *P* values were adjusted using the Benjamini and Hochberg’s approach for controlling the false discovery rate. Genes with|log2 (Fold Change)|> 0 & padj < 0.05 were assigned as differentially expressed^52^.

### lncRNAs target gene prediction

Target gene prediction of lncRNAs was carried out in two ways: the cis-acting target gene prediction, and trans-acting target gene prediction. Based on the theory of cis-acting regulatory element, the protein-coding genes located within 10/100 kb from lncRNA were selected as potential cis-acting target. While for trans-acting target prediction, the Pearson’ correlation coefficients between the coding genes and lncRNAs were calculated and analyzed for the identification of trans-acting regulatory elements, which required a sample size more than five.

### KEGG enrichment analysis

KEGG enrichment analysis of target genes of differentially expressed lncRNAs was implemented by the cluster Profiler R package, in which gene length bias was corrected. The enrichment was considered to be significant when the corrected *p* values were less than 0.05. KEGG enrichment analysis was carried out using KOBAS (2.0)^53,54^.

### LncRNAs-mRNAs co-expression network analysis

The analysis of interactions between lncRNAs and mRNAs was mainly based on the co-expression results of lncRNAs and mRNAs. For each lncRNA-mRNA pair, the Pearson correlation coefficient (PCC) was calculated and for those with PCC > 0.95 were applied for further differential expression analysis^55^. Cytoscape software (http://www.cytoscape.org/) was applied for presenting the co-expression networks of lncRNAs-mRNAs interactions.

### Validation of sequencing result by qRT-PCR

Then, 0.5 μg of RNA was reverse transcribed to cDNA in a volume of 20 μl reaction using random primer and PrimeScript reverse transcriptase according to the manufacturer’s instructions.

After reverse transcription reaction, 1 μl of reaction mixture was proceeded to PCR reaction using rTAQ (94 °C for 3 min; 40 cycles of 94 °C for 30 s, 60 °C for 30 s, and 72 °C for 30 s ; 72 °C for 2 min) using specific primer pairs:

*GAPDH* forward: 5’-GACCACAGTCCATGCCATCACT-3’

and reverse 5’-TCCACCACCCTGTTGCTGTAG-3’;

*Fgf18* forward: 5’-TGC GCT TGT ACC AGC TCT AC-3’

and reverse 5’-CAC TCC TTG CTA GTA CCA TC-3’;

*Vcam1* forward: 5’-ATG TGC TGC TGT TGG CTG TGA CTC-3’

and reverse: 5’-GGC TCA GCG TCA GTG TGG ATG TAG-3’;

*Pck2* forward: 5’-TGTCATCCGCAAGCTGAAGAA-3’

and reverse 5’-GCTTTCGATCCTGGCCACAT-3’;

ENSRNOT00000084874, forward: 5’-GAGGCCTTGTCCTGTTCCTT-3’;

and reverse 5’- CGTACCTGCTGCCATTCCTT -3’;

ENSRNOT00000082515, forward: 5’- CAATGTCTGCCACCTACTGATC -3’;

and reverse 5’- TGTGAGGGAGGAAGGGAAC -3’;

LNC_001033 forward:5’- GCCTGCAGAGGAAAGCAATG -3’;

and reverse 5’- TATCTGTGGGGTCAGGGGAG -3’.

Transcript levels of ENSRNOT00000084874, ENSRNOT00000082515, LNC_001033 forward *Fgf18*, *Vcam1*, and *Pck2* were measured by quantitative real-time PCR using PerfectStartTM Green qPCR SuperMix (Transgene, Beijing, China) and ABI 7500 system.

In each reaction, 2 μl of cDNA from each sample was added after 5 × dilution. For each primer pair, PCR efficiency was determined by melting curve and the transcript levels were normalized against *GAPDH*, and calculated 2^−ΔΔCT^ value for each group. Quantitative real-time PCR experiments were independently performed three times. All of primers were synthesized by Sangon Biotechnology, Shanghai, China.

### Statistical analysis

The results shown are from at least three independent experiments. Data were analyzed for statistical significance using one-way ANOVA and Student’s *t*-test. Differences were considered significant at *p* < 0.05 as indicated.

### Ethical approval

This study does not contain any studies with human participants or animal performed by any of the authors.

## Supplementary Information

Supplementary Information 1.

Supplementary Information 2.

Supplementary Information 3.

Supplementary Information 4.

Supplementary Information 5.

Supplementary Information 6.

Supplementary Information 7.

Supplementary Information 8.

Supplementary Information 9.

Supplementary Information 10.

## Data Availability

The data for Novel lncRNAs found in PC12 cells in this study has been deposited in NCBI gene bank with BioProject accession number accession number PRJNA718985. The data that support the findings of this study are also available from the corresponding author upon reasonable request.
